# Aging affects sex- and organ-specific trace element profiles in mice

**DOI:** 10.18632/aging.103572

**Published:** 2020-07-03

**Authors:** Kristina Lossow, Johannes F. Kopp, Maria Schwarz, Hannah Finke, Nicola Winkelbeiner, Kostja Renko, Xheni Meçi, Christiane Ott, Wiebke Alker, Julian Hackler, Tilman Grune, Lutz Schomburg, Hajo Haase, Tanja Schwerdtle, Anna P. Kipp

**Affiliations:** 1 Department of Molecular Nutritional Physiology, Institute of Nutritional Sciences, Friedrich Schiller University Jena, Jena, Germany; 2 Department of Food Chemistry, Institute of Nutritional Science, University of Potsdam, Nuthetal, Germany; 3 German Institute of Human Nutrition, Nuthetal, Germany; 4 TraceAge-DFG Research Unit on Interactions of Essential Trace Elements in Healthy and Diseased Elderly, Potsdam-Berlin-Jena, Germany; 5 Institute for Experimental Endocrinology, Charité University Medical School Berlin, Berlin, Germany; 6 German Federal Institute for Risk Assessment (BfR), Berlin, Germany; 7 DZHK German Centre for Cardiovascular Research, Berlin, Germany; 8 Department of Food Chemistry and Toxicology, Technische Universität Berlin, Berlin, Germany

**Keywords:** aging, trace elements, biomarkers, homeostasis, epigenetic markers

## Abstract

A decline of immune responses and dynamic modulation of the redox status are observed during aging and are influenced by trace elements such as copper, iodine, iron, manganese, selenium, and zinc. So far, analytical studies have focused mainly on single trace elements. Therefore, we aimed to characterize age-specific profiles of several trace elements simultaneously in serum and organs of adult and old mice. This allows for correlating multiple trace element levels and to identify potential patterns of age-dependent alterations. In serum, copper and iodine concentrations were increased and zinc concentration was decreased in old as compared to adult mice. In parallel, decreased copper and elevated iron concentrations were observed in liver. The age-related reduction of hepatic copper levels was associated with reduced expression of copper transporters, whereas the increased hepatic iron concentrations correlated positively with proinflammatory mediators and Nrf2-induced ferritin H levels. Interestingly, the age-dependent inverse regulation of copper and iron was unique for the liver and not observed in any other organ. The physiological importance of alterations in the iron/copper ratio for liver function and the aging process needs to be addressed in further studies.

## INTRODUCTION

Aging is an inevitable biological process with concomitant changes on the cellular level, including mitochondrial dysfunction, genomic instability, epigenetic alterations, protein aggregation, telomere attrition, and cellular senescence [1]. The underlying mechanisms for the observed changes are yet to be fully identified, however, several aging hypotheses are based on an increase in oxidative stress [2–4] and concomitant accumulation of oxidized and nitrated proteins [5–7], oxidized lipids, and DNA damage [8]. One contributor to the increase in oxidative stress is supposed to be the age-dependent decline of the nuclear factor (erythroid-derived 2)-like 2 (Nrf2) responsiveness [9–11]. Nrf2 controls the transcription of antioxidant, cytoprotective, and detoxification genes, including NAD(P)H:quinone oxidoreductase (*NQO1*), several glutathione S-transferase isoforms (*GST*, isoforms a1, a2, a3, a5, m1, m2, m3, p1) [12], as well as genes involved in glutathione (GSH) synthesis [13]. Furthermore, genes related to trace element (TE) metabolism such as ferritin H (FTH) are regulated via Nrf2 [14, 15]. Besides oxidative stress, functional impairment of the immune response and a systemic chronic low-grade inflammation, referred to as “inflammaging”, are hallmarks of the aging process [16–20]. Low-grade inflammation is characterized by enhanced constitutive circulation of inflammatory mediators such as cytokines, e.g., interleukin 6 (IL6), tumor necrosis factor-alpha (TNFα), and acute-phase proteins [21, 22], in the absence of clinically defined infections.

During the aging process, essential TEs are important as they modulate both oxidative stress and immune response by their indispensable functions, e.g., in enzymatic reactions and signaling pathways. Elderly subjects are prone to inadequate TE intake [23, 24], which results in lower serum concentrations of e.g., selenium (Se) [25] and zinc (Zn) [26]. This is particularly worrisome for Se because its supply is already suboptimal in the general European population [27]. A low Se status is associated with an increased risk of infections, cancer, other age-related diseases, and mortality [28]. Similarly, the immune response is impaired by a low Zn status, especially under conditions of chronic inflammation [29]. However, other TEs such as copper (Cu) are increased in the elderly [30]. So far, most studies investigated single TEs only. In our hypothesis, interactions of TEs might contribute to their age-related changes, thereby generating age-specific TE patterns. The competition of Cu and Zn for intestinal absorption and metallothionein (MT) binding is an instructive example [31]. Because of this interaction, it is assumed that the Cu/Zn ratio is a more conclusive parameter than a separate analysis of both elements [26].

We aim to systematically extend this concept by considering six essential and health-relevant TEs in parallel, namely Cu, iodine (I), iron (Fe), manganese (Mn), Se, and Zn. To this end, age-related TE profiles are determined in serum and several organs of adult versus old C57BL/6Jrj mice and correlated with parameters of aging, e.g., nitrated proteins, epigenetic modifications, inflammatory mediators, and Nrf2 target genes. This will provide the basis for further studies concerning the underlying mechanisms of age-related shifts in TE profiles and the relevance of these changes for age-related characteristics and diseases.

## RESULTS

To determine the TE status, *ad libitum* chow-fed animals of both sexes were sacrificed at the age of 24 (adult) or 109 to 114 weeks (old). Male mice showed no age-dependent differences in body weight (Supplementary Table 1). In contrast, the body weight of female mice was significantly increased in old mice. Generally, females had a significantly lower body weight as compared to males (Supplementary Table 1). Relative organ weights were largely unaffected by age, with the exception of relative heart and kidney weights, which significantly increased with age (Supplementary Table 1). The vast majority of old mice developed dysfunctions. In particular, a high incidence of splenomegaly and tumors primarily affecting mesentery and intestine was detected.

### Age- and sex-dependent changes of TE concentrations in serum

**Figure 1:**
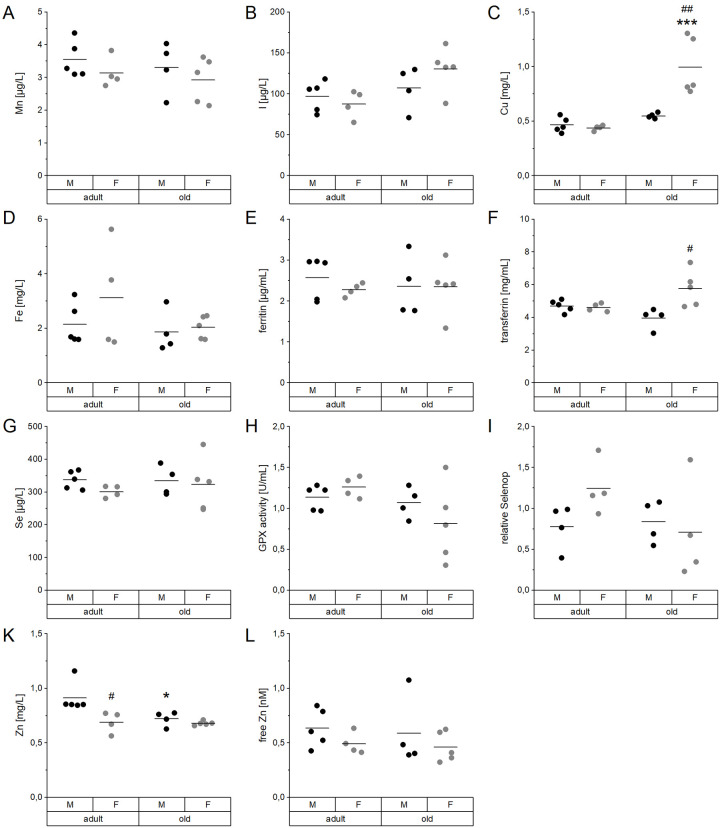
**Age- and sex-related changes of serum TE profiles and biomarkers.** Concentrations of Mn (**A**), I (**B**), Cu (**C**), Fe (**D**), Se (**G**), and Zn (**K**) were analyzed in the serum of adult (24 weeks) and old (109-114 weeks) male and female C57BL/6Jrj mice (n = 4-5) receiving chow diet. Serum concentrations were determined using ICP-MS/MS (**A**-**D**, **G**, **K**). Further biomarkers were detected by ELISA (**E**, **F**) and fluorescent probes (**L**) to assess the Fe marker ferritin (**E**) and transferrin (**F**) as well as free Zn (**L**), respectively. The Se status was further validated by GPX activity (**H**) and relative Selenop levels (**I**), based on NADPH-consuming glutathione reductase coupled assay and Dot blot analysis, respectively. Statistical testing based on Two-Way ANOVA and post hoc analysis using Bonferroni’s test with * p < 0.05, *** p < 0.001 vs. adult and ^#^ p < 0.05, ^##^ p < 0.01 vs. male.

In serum, concentrations of Cu, I, Fe, Mn, Se, and Zn as well as functional biomarkers for Fe, Se, and Zn were determined (Figure 1, Supplementary Table 2). No significant differences between male and female mice or both age groups were detected for Mn and I (Figure 1A, 1B). However, serum concentrations of I showed an age-dependent increase when considering all mice irrespective of their sex (Supplementary Table 2). Serum Cu levels were significantly increased in old female mice, both in comparison to young females and old male mice (Figure 1C). Fe and ferritin serum levels were not altered in the mouse cohort (Figure 1D, 1E), while transferrin was significantly increased in aged females in comparison to aged male mice (Figure 1F). The average Se concentration (Figure 1G) as well as the levels of the selenoprotein-based functional biomarkers GPX activity (Figure 1H) and selenoprotein P (Selenop) (Figure 1I) were unaffected by age or sex. Serum Zn concentrations were decreased in old male and adult female mice, compared to adult males (Figure 1K). However, free Zn, often used as an alternative status marker, stayed the same (Figure 1L). Spearman’s correlation analysis (Supplementary Table 3) revealed strong positive correlations between Cu and I (r_S_=0.701, p=0.001) as well as Zn and Se serum concentrations (r_S_=0.509, p=0.031). Relative Selenop protein levels were negatively correlated with serum I concentrations (r_S_= 0.662, p=0.005).

### TE profiles in murine organs

**Figure 2:**
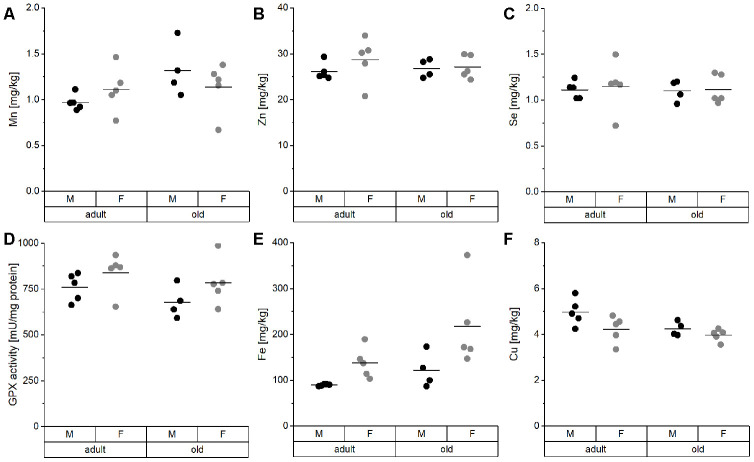
**TE profile analysis in the liver of mice.** Liver tissue of adult (24 weeks) and old (109-114 weeks) male and female C57BL/6Jrj mice (n = 4-5) receiving chow diet were analyzed for their concentrations of Mn (**A**), Zn (**B**), Se (**C**), Fe (**E**), and Cu (**F**) using ICP-MS/MS. Furthermore, Se-sensitive GPX activity was assessed by NADPH-consuming assay (**D**). Statistical testing based on Two-Way ANOVA and post hoc analysis using Bonferroni’s test revealed no significant differences for age and sex.

**Figure 3:**
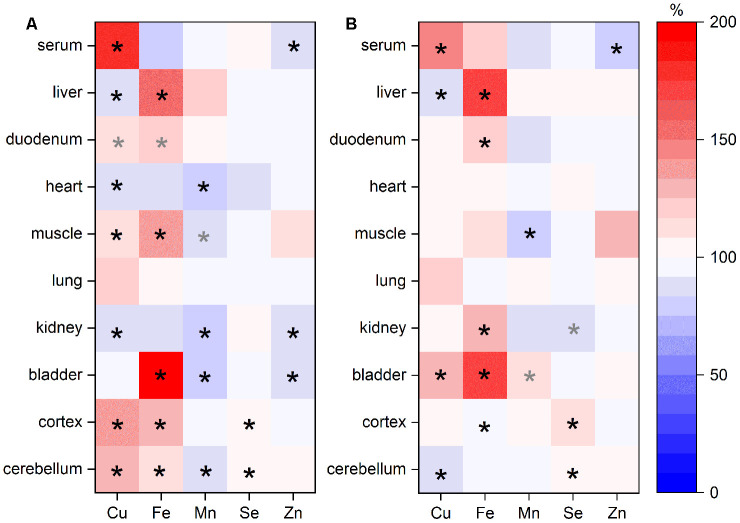
**TE changes in various organs in relation to age and sex.** TEs in various organs of adult (24 weeks) and old (109-114 weeks) male and female C57BL/6Jrj mice receiving a chow diet *ad libitum* were analyzed by ICP-MS/MS. The heat map indicates changes of TE content in murine organs of old mice compared to adult animals (**A**; n=9-10) or of female mice in comparison to male animals (**B**; n=9-10) given in % (100 % represents no change). Each row represents one organ, whereas each column represents one element. Statistical testing based on Two-Way ANOVA and post hoc analysis using Bonferroni’s test with * p < 0.05, whereas grey * indicates p < 0.1.

TE concentrations in the liver did not show any significant difference between groups (Figure 2). Mn, Zn, and Se concentrations, as well as hepatic GPX activity, were entirely stable in all groups (Figure 2A–2D). Only Fe and Cu concentrations showed a trend toward upregulation of Fe and downregulation of Cu in old mice (Figure 2E, 2F), which was significant when considering all mice irrespective of sex (Supplementary Table 2). Thus, aging affects Cu levels in opposite directions in serum and liver (Figure 3).

Besides serum and liver, the distribution of Cu, Fe, Mn, Se, and Zn was further assessed in duodenum, heart, muscle, lung, kidney, bladder, cortex, and cerebellum. Across these organs, we observed profoundly different distribution patterns for the analyzed TEs (Figure 3, Supplementary Table 2). Overall, Se and Zn levels in organs were relatively stable across the age groups. If Zn concentrations were altered, they were downregulated in old mice. In most organs of old mice, Mn was also consistently downregulated. In contrast, Fe was upregulated showing the highest fold change in bladder. Cu was decreased in liver, heart, and kidney of old mice but increased in all other organs, with exception of the bladder (Figure 3A). Sex-related differences were observed for Fe with higher levels in females (Figure 3B).

### Putative interactions of TEs based on correlation analyses

TE interactions between and within the organs were evaluated based on analysis of Spearman’s correlation coefficients (Supplementary Tables 3, 4; Supplementary Figure 1). Most often, Cu serum concentrations correlated positively with Fe concentrations in organs, e.g., in the liver. In contrast, serum Cu levels correlated negatively with Mn levels in diverse organs. Negative correlations were also observed for serum Zn and Fe concentrations in organs. Within the same organ, Cu concentrations correlated with Fe, Mn, and Se in a positive manner in most cases. Also, Se and Zn concentrations correlated positively in multiple organs, e.g., liver or kidney. While Se and Mn levels showed positive correlations in heart and kidney, a negative correlation was obtained in the cerebellum.

### Hepatic expression of genes related to uptake and distribution of TEs

**Figure 4:**
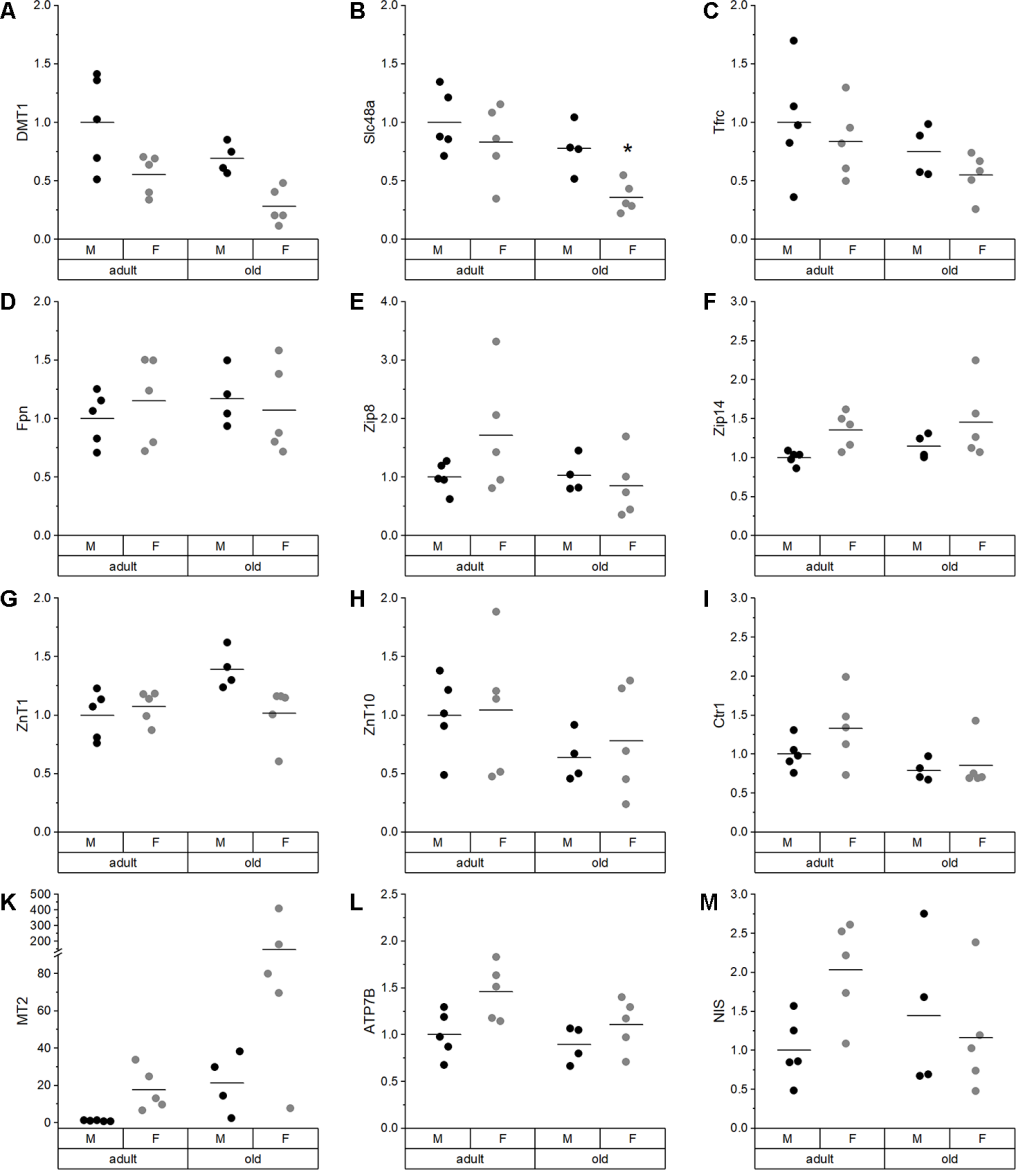
**Expression analysis of various TE-related genes in liver.** Relative expression levels of TE-related genes in the liver of adult (24 weeks) and old (109-114 weeks) male and female mice (n = 4-5) fed with a chow diet *ad libitum*. Expression levels were normalized by a composite factor based on the house-keeping genes Hprt and Rpl13a. Finally, variances are expressed as fold change compared to adult males (mean adult males = 1). Statistical testing based on Two-Way ANOVA and post hoc analysis using Bonferroni’s test with * p < 0.05.

In order to identify putative mechanisms for the observed TE distribution patterns, mRNA expression analyses for various transport and binding proteins involved in the cellular transfer and storage of Cu, I, Fe, Mn, and Zn were performed in duodenum and liver (Supplementary Figure 2; Figure 4). In the duodenum, expression levels of the Cu exporter ATPase copper transporting alpha (ATP7A) were downregulated in old females in comparison to adult females (Supplementary Figure 2M). In contrast, the Cu- and Zn-binding proteins metallothionein 1 (MT1) and MT2 (Supplementary Figure 2K, 2L) were upregulated with increasing age in males, whereas females tended toward the opposite effect.

Hepatic proton-coupled divalent metal ion transporter (DMT1), involved in the uptake of divalent metals such as Cu, Fe, Mn, and Zn tended to be reduced in old as compared to adult mice, with lower expression levels in female mice (Figure 4A). Similar changes as for DMT1 were observed for the heme transporter solute carrier family 48 member 1 (Slc48a, Figure 4B) and transferrin receptor (Tfrc, Figure 4C). The latter showed a trend for lower expression levels with age. The expression levels of the Fe exporter ferroportin (Fpn, Figure 4D) and solute carrier family 39, member 8 (Zip8, Figure 4E), responsible for the influx of Zn, Mn, and Fe [32], were unaffected. Zip14, which is involved in the hepatic uptake of Zn, Mn, and Fe, showed a trend for higher expression levels in females, which was independent of age (Figure 4F). Solute carrier family 30, member 1 (ZnT1), responsible for Zn export, tended to be upregulated in old male mice in comparison to adult males (Figure 4G), whereas ZnT10 was unaffected (Figure 4H). The Cu transporter solute carrier family 31, member 1 (Ctr1) showed a trend for downregulation in old mice (Figure 4I). Hepatic MT2 expression tended toward an age-dependent increase in both males and females (Figure 4K). For the hepatic Cu-transporting ATPase, ATPase copper transporting beta (ATP7B), a trend for higher expression in females was observed (Figure 4L). The sodium iodide symporter NIS did neither change with age nor sex (Figure 4M).

Whereas the expression levels of these TE-related genes did not strongly correlate with TE concentrations in serum, liver, or duodenum, highly significant correlations between the individual transporters were identified (Supplementary Figure 3). Especially TE-related genes associated with Cu, Zn, and Fe uptake or export were strongly correlated.

### Age-dependent effects on mediators of inflammation, DNA methylation, nitrated proteins, and Nrf2 target genes

**Figure 5:**
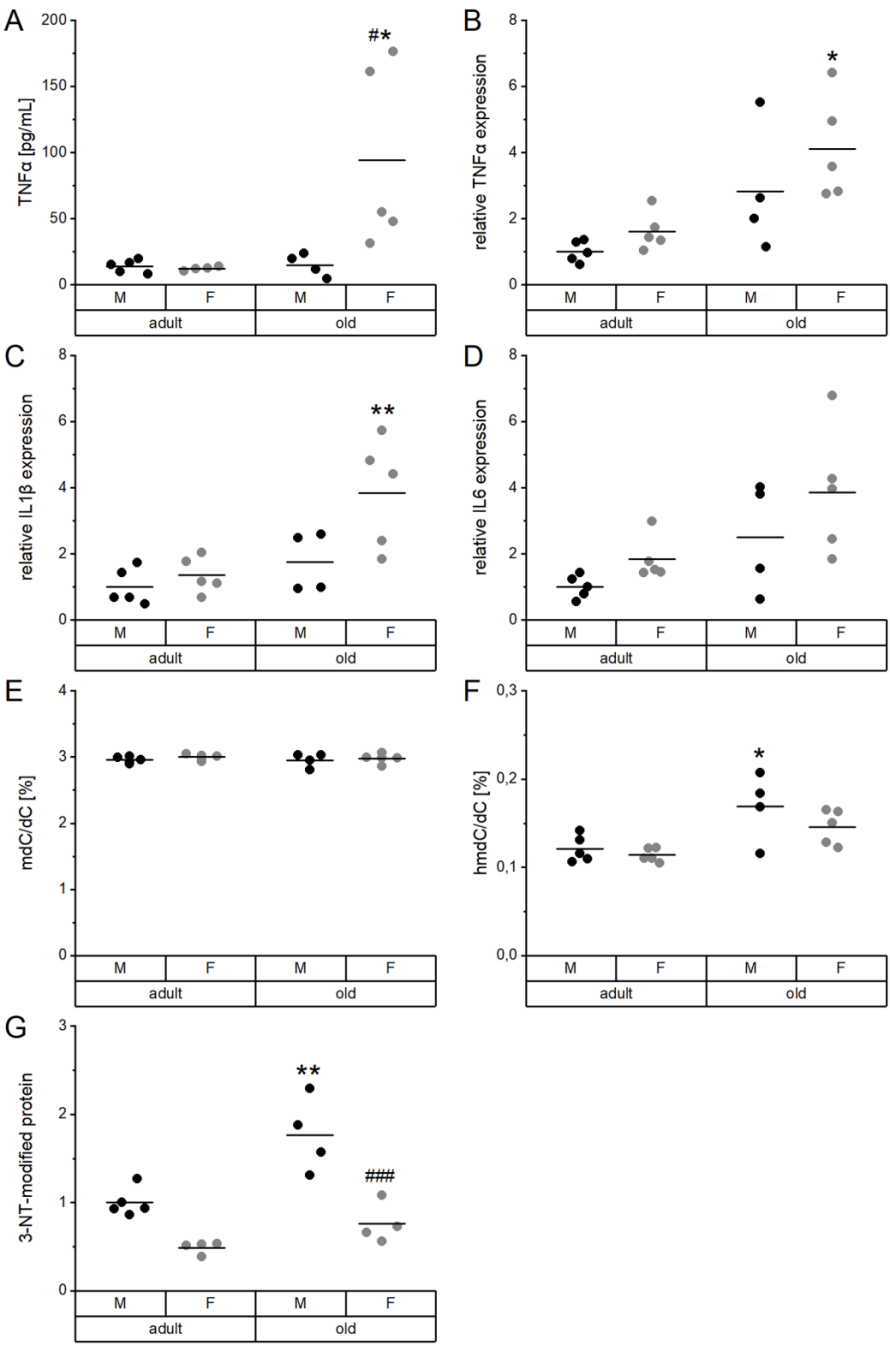
**Proinflammatory cytokines and DNA and protein modifications in relation to age and sex.** Serum (**A**) and liver extracts (**B**–**G**) of adult (24 weeks) and old (109-114 weeks) male and female mice (n = 4-5) fed with a chow diet *ad libitum* were subjected to enzyme-linked immunosorbent assay (**A**), qRT-PCR analysis (**B**–**D**), tandem mass spectrometry (**E**, **F**), and immunoblotting (**G**). This way, proinflammatory cytokines (**A**–**D**), global DNA methylation (mdC/dC; **E**), and hydroxymethylation (hmdC/dC; F), as well as 3-nitrotyrosine (3-NT, G) protein modifications were determined. Hepatic transcription levels (**B**–**D**) were normalized by a composite factor based on the house-keeping genes Hprt and Rpl13a, whereas 3-NT-modified proteins were normalized to GAPDH (**G**). Except for (**E**) and (**F**), where data is given in %, data is presented as fold change compared to male adults (**A**–**D**, **G**). Statistical testing based on Two-Way ANOVA and post hoc analysis using Bonferroni’s test with * p < 0.05, ** p < 0.01 vs. adult and ^#^ p < 0.05, ^###^ p < 0.001 vs. male.

Inflammatory mediators, DNA methylation, as well as protein modifications were determined to characterize age-related changes in the mouse cohort and to correlate these to the TE profiles. In serum, TNFα levels were significantly increased in old female mice as compared to adult females as well as to old males (Figure 5A). A comparable increase in old female mice was observed for the hepatic mRNA expression levels of TNFα (Figure 5B), IL1β (Figure 5C), and IL6 (Figure 5D). TNFα levels in serum were strongly correlated with serum Cu levels (Supplementary Table 3), while all hepatic proinflammatory cytokines were correlated with Fe concentrations in liver (Supplementary Table 4, Supplementary Figure 1).

Global DNA methylation (mdC/dC) in the liver was neither affected by age nor sex (Figure 5E), whereas DNA hydroxymethylation (hmdC/dC) was significantly enhanced in old male mice (Figure 5F). Hepatic levels of tyrosine nitrated proteins (3-NT) were also increased in old male mice compared to adult males. Levels were generally lower in females than in males (Figure 5G). DNA hydroxymethylation correlated with 3-NT-modified proteins but not with the hepatic concentration of any of the TEs (Supplementary Table 4, Supplementary Figure 1).

**Figure 6:**
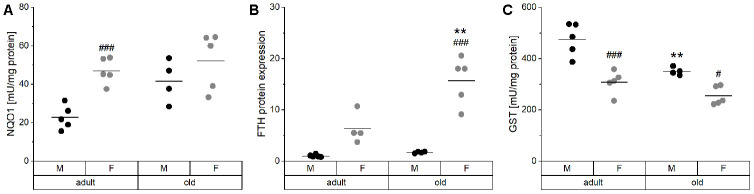
**Activity and expression levels of Nrf2 target genes.** The enzyme activities of the Nrf2 targets NQO1 (**A**) and total GST (**C**) were determined by activity assays, whereas the relative protein levels of FTH (**B**) normalized to the house-keeping gene GAPDH were analyzed by Western blot in liver tissue of adult (24 weeks) and old (109-114 weeks) male and female mice (n = 4-5). Statistical testing based on Two-Way ANOVA and post hoc analysis using Bonferroni’s test with ** p < 0.01 vs. adult and ^#^ p < 0.05, ^###^ p < 0.001 vs. male.

Hepatic Nrf2 target genes were analyzed, including NQO1 activity, FTH expression, and total GST activity (Figure 6). Especially in male mice, NQO1 activity tended to increase with age and showed higher overall values in females (Figure 6A). The same age-dependent increase was observed in heart, lung, and kidney of male mice (Supplementary Figure 4). FTH levels revealed a similar pattern as NQO1 activity with higher levels in females and an age-dependent increase (Figure 6B). Both hepatic NQO1 activity and FTH expression correlated positively with the expression of inflammatory mediators and with Fe concentrations in the liver (Supplementary Table 4, Supplementary Figure 1). Interestingly, hepatic GST activity behaved in an opposite manner being decreased with age (Figure 6C) and increased in male mice. However, these effects on GST were not detected in other organs (Supplementary Figure 4).

## DISCUSSION

The old mouse cohort studied herein displayed typical characteristics described for old organisms. This includes an increase in relative heart weight (Supplementary Table 1), typical for a hypertrophic, aging heart [33–35], and an increase in kidney weight (Supplementary Table 1), which was previously observed in old rodents [36]. Biomarkers of aging include 3-NT modified proteins [37, 38], which were increased with age in livers of male mice (Figure 5G). In addition, we observed higher global levels of the oxidation product 5-hmdC in the liver of old mice (Figure 5F), which is in line with previously published results [39]. On the contrary, global DNA methylation seems to be less predictive for age [40–45], which was confirmed in this study for liver (Figure 5E).

Most of the observed age-dependent differences in serum TE profiles (Figure 1) are in line with published values for single TEs, but now provide an overall picture. The data allow for correlating multiple TE concentrations and to identify potential patterns of age-dependent alterations. Most pronounced, Cu concentrations in serum increased with age, especially in female mice (Figure 1C), as has been reported before in rodents and in humans [46–50]. Serum Zn levels behaved the opposite (Figure 1K), which is in line with the literature for several species [29, 50–54]. However, free Zn, which is discussed to be a more reliable biomarker for bioavailable Zn [55], was not affected by age (Figure 1L). Additionally, several human studies reported a significant increase of I and Fe and a reduction of Se concentrations in the serum of elderly subjects [50, 52, 56]. In the present study, a tendency toward increased serum I levels was observed (Figure 1B). However, a more reliable biomarker is urinary iodine concentration at least in human cohorts [57, 58]. As urine samples were not available for our mice, we additionally analyzed the I content of the thyroid which was rather heterogenous in old mice but tended to increase with age (Supplementary Figure 5A). In addition, a significant reduction of the enzymatic activity of the I-releasing deiodinase I (Dio1) was observed (Supplementary Figure 5B). This indicates age-related changes of iodine storage and handling, that would need further clarification in future studies. Concerning Fe, the only biomarker that was upregulated in old female mice was serum transferrin (Figure 1F), while serum ferritin was unaffected (Figure 1E). Serum transferrin was shown to correlate with hepatic Fe levels but not with serum Fe [59], which was also the case in this study. Neither serum Se levels, nor the functional biomarkers GPX activity or Selenop levels were affected by age (Figure 1G–1I). A previous study using telomerase RNA component knockout mice reported marginally increased Se and Selenop levels with age in the plasma of male but not female mice [60].

In the liver, which was the organ with the highest TE concentrations in this study (Supplementary Table 2; [61]), we observed a trend toward higher Fe levels in old than in adult mice (Figure 2E) together with reduced hepatic Cu levels (Figure 2F). This has been previously described for individual TEs during aging [23, 62–64], but has not been shown to be linked to each other. Strikingly, this relationship was unique for the liver. In most other organs, significant positive correlations between Fe and Cu were observed (Supplementary Figure 1). To understand the underlying mechanisms of age-dependent changes in hepatic TE concentrations, expression levels of transport and binding proteins were analyzed. DMT1, a transporter shared by Cu and Fe [65], was downregulated in old mice (Figure 4A), which provides an explanation for lower hepatic Cu levels in old mice. Besides DMT1, Cu ions are mainly transported by Ctr1, which was downregulated with age as well (Figure 4I). ATP7B expression also tended to be downregulated in old mice (Figure 4L), thus enhanced Cu export from liver does most probably not contribute to lower hepatic Cu levels. Other studies have, however, not detected any age-related change in net Cu absorption [66]. Serum Fe is mainly bound to transferrin and taken up by a Tfrc-mediated mechanism, but Tfrc expression was unaffected by age (Figure 4C). The same was the case for the Fe exporter Fpn (Figure 4D). Based on these observations, age-related restrictions of hepatic Cu levels were associated with lower expression levels of transporters, while the increase in hepatic Fe concentrations appears to depend on other mechanisms.

TEs contribute to immune function in many different ways [67–70]. *Vice versa*, a low-grade chronic inflammation frequently observed in the elderly might contribute to the observed age-specific shifts in TE profiles [51]. Indeed, Zn concentrations in serum decline during acute inflammatory disorders or infections [71–76], as well as in aged animals, as shown herein (Figure 1K) or for elderly humans [50]. These effects are supposed to be mainly mediated by IL1 and IL6 [77–79]. Furthermore, we detected an age-dependent increase in Cu serum concentrations (Figure 1C, Supplementary Table 2), which also takes place under inflammatory conditions [72, 74, 75]. Also, Fe is a negative acute-phase reactant [80–82], which is modulated by enhanced secretion of hepcidin [83] and stimulated by cytokines such as IL6. Hepcidin interacts with Fpn, limiting the Fe release from cells into the bloodstream [84]. Fe concentrations were indeed increased in several organs of old mice, including the liver (Supplementary Table 2), and hepatic Fe concentrations correlated with inflammatory mediator expression in the liver. Thus, low-grade inflammation appears to be the main mechanism for age-related Fe accumulation in the liver.

The age-related overproduction of pro-inflammatory cytokines not only causes a low-grade inflammation, but also shifts the cellular redox state, thereby contributing to constitutive activation of the Nrf2 system. Effectively, this higher constitutive Nrf2 activity is the reason for reduced Nrf2 responsiveness in old organisms [85–87]. As TE-related proteins like Fpn, hepcidin, or ferritin [88, 89] are regulated via Nrf2, this provides another potential mechanism for age-related modifications of the TE patterns, especially for alterations of the Fe status. A significant age-dependent increase in NQO1 activity was observed in all organs analyzed (Figure 6A, Supplementary Figure 4). In addition, higher amounts of FTH were detected in liver tissue of old mice (Figure 6B) and correlated strongly with hepatic Fe concentrations. Overall, the Nrf2-induced increase of FTH protein expression may provide an explanation for higher hepatic Fe levels (Figure 6B). Accordingly, inflammatory processes and/or increased Nrf2 activity may constitute the driving forces for the age-specific alterations in TE concentrations observed in serum and liver.

In summary, aging is associated with profound differences in TE concentrations in serum and different organs. The parallel analysis of six TEs highlights particular alterations in serum TE profiles of old versus adult mice, with decreased Zn and increased Cu and I concentrations. The reciprocal alterations in serum Cu and Zn concentrations were, however, not observed in the analyzed organs. A second organ-specific effect was detected in liver, where we observed an age-dependent inverse regulation of Cu and Fe concentrations. While these changes in the Fe/Cu ratio may be directly related to systemic low-grade inflammation, their physiological importance for liver function and the aging process needs to be addressed in further studies.

## MATERIALS AND METHODS

### Animal experiment

Male and female C57BL/6Jrj mice were housed on a 12:12 h light:dark schedule with food and tap water *ad libitum*. We utilized a commercially available chow diet (V1534, Ssniff, Soest, Germany) with Fe, Zn, Mn, Cu, I, and Se content of 215, 97, 82, 8.8, 1.8, and 0.3 mg/kg diet, respectively. It needs to be considered that the TE amounts in the diet exceeded the nutritional TE requirements for mice [90]. While Se and Cu were marginally increased (2-fold and 1.5-fold above the requirement, respectively), the other four TEs had levels 12-fold for I, 10-fold for Zn, 8-fold for Mn, and 6-fold for Fe above the respective requirements. Tap water contained about 0.03, 0.35, 0.66 mg/L Fe, Zn, and Cu, respectively. Mn and Se content of drinking water was about 0.75 and 0.03 μg/L, respectively.

At the age of either 24 weeks (adult) or 109-114 weeks (old), mice were anesthetized with isoflurane (Cp-pharma, Burgdorf, Germany), and blood was collected by cardiac puncture. Serum was obtained after full coagulation at room temperature (RT) and centrifugation for 10 min (2,000 x g, 4°C). Organs were surgically dissected and immediately frozen. All animal procedures were approved and conducted following national guidelines of the Ministry of Environment, Health and Consumer Protection of the federal state of Brandenburg (Germany, 2347-44-2017) and institutional guidelines of the German Institute of Human Nutrition Potsdam-Rehbruecke.

### ICP-MS/MS analysis of trace elements in serum, organs, and feed

TEs in the serum were determined as described previously [91, 92]. In brief, 50 μL of murine serum were diluted 1:10 with a dilution mix (5 % (v/v) butanol (99 %, Alfa Aesar, Karlsruhe, Germany), 0.05 % (w/v) Na-EDTA (Titriplex® III, pro analysis, Merck, Darmstadt, Germany), 0.05 % (v/v) Triton™ X-100 (Sigma-Aldrich/Merck, Taufkirchen, Germany) and 0.25 % (v/v) ammonium hydroxide (puriss. p.a. plus, 25 % in water, Fluka, Buchs, Germany)) as well as internal standards (final concentrations: 1 μg rhodium (Rh)/L and 30 μg ^77^Se/L). Cu, I, Fe, Mn, Se (IDA), and Zn concentrations were determined in the diluted sample using ICP-MS/MS (8800 ICP-QQQ-MS, Agilent Technologies, Waldbronn, Germany).

20-50 mg of snap-frozen organs (unless the total weight of the organ was lower than 50 mg, in which case organs were digested as a whole) or feed were homogenized using mortar and pestle under liquid nitrogen or at RT, respectively, and were weighed into PTFE microwave vessels. HNO_3_ (65 %, Suprapure®, Merck) and H_2_O_2_ (30 %, Sigma-Aldrich/Merck) were used for digestion. Additionally, Rh (diluted from 10 mg/L single element stock solution, Carl Roth, Karlsruhe, Germany) and ^77^Se (diluted from a 10.000 mg/L stock solution, prepared in house from isotopically enriched ^77^Se (97.20 ± 0.20 % ^77^Se; 0.10 % ^74^Se; 0.40 ± 0.10 % ^76^Se; 2.40 ± 0.10 % ^78^Se; 0.10 % ^80^Se; 0.10 % ^82^Se as certified by Trace Sciences International, Ontario, Canada), purchased from Eurisotop SAS (Saarbrücken, Germany) were added as internal standard and isotope dilution standard, respectively. The samples were digested in a MARS 6 microwave digestion system (CEM, Kamp-Lintfort, Germany) by heating to 200°C over 10 min and holding this temperature for 20 min. Post digestion, the samples were diluted with ultrapure water to give final concentrations of 2.93 % HNO_3_, 10 μg/L ^77^Se, and 1 μg/L Rh. This solution was subjected to ICP-MS/MS analysis with the following parameters: 1550 W plasma Rf power, Ni-cones, MicroMist nebulizer at 1.2 L Ar/min and Scott-type spray chamber). The following mass to charge ratios and gas modes were used (Q1→Q2): He-mode: Mn (55→55), Fe (56→56), Cu (63→63), Zn (66→66), Rh (103→103); O2-mode: Se (77→93), Se (80→96), Rh (103→103). Elements in He-mode were determined via external calibration using calibration solutions made from 1000 mg/L single element standard solutions (Carl Roth) with internal standard correction using Rh. Se was determined via IDA, as described previously [93]. Results were checked using certified reference materials ERM-BB 422 (fish muscle) or ERM-BB 186 (pig kidney), and the analysis was repeated if reference material recovery deviated by more than 10 % from the certified value. To preserve animal material for the analysis of other markers, the variability of three independent digestions was checked for liver tissue in another animal experiment and found to be below 5 %. Therefore, in subsequent experiments, each organ was analyzed as a single replicate, unless extreme outliers as compared to animals from the same experimental group were identified, in which case the analysis was repeated.

### Analysis of free zinc in serum

Free zinc was determined by the low molecular weight fluorescent probe Zynpyr-1, as reported before [94]. For the application on murine serum samples, the assay was modified as follows: The incubation times for F, F_min_, and F_max_ were set to 60, 30, and 60 min, respectively. For the induction of F_min_ and F_max_, 15 μL EDTA solution (800 μM) or ZnSO_4_ solution (25 mM) per well were added, resulting in final concentrations of 100 μM EDTA and 2.8 mM ZnSO_4_, respectively.

### ELISA for ferritin and transferrin

The concentrations of ferritin and transferrin were determined using an ELISA kit (ALPCO, Salem, USA) according to the manufacturer's instructions. Briefly, serum was diluted either 1:20 or 1:200,000 for ferritin and transferrin analysis, respectively. A volume of 100 μL (standard, control, or sample) was added to the wells. After incubation for 60 and 30 min at RT, wells were washed, and 100 μL of horseradish peroxidase-anti-ferritin or -anti-transferrin conjugate was added, respectively. Following a further incubation at RT, 100 μL of chromogenic substrate solution was added. After 10 min, the enzymatic reaction was stopped by adding 100 μL stop solution. The optical density was determined at 450 nm using an Infinite 200 Pro microplate reader (Tecan, Männedorf, Switzerland). Average absorbance was determined and concentrations calculated based on standard curves.

### TNFα analysis in serum

TNFα protein in serum was quantified by ProQuantum Mouse TNFα Immunoassay Kit (Invitrogen, ThermoFisher Scientific, Waltham, MA, USA). Briefly, serum was diluted 1:3 and subjected to an equal volume of the antibody-conjugated mixture on the assay plate. The antibodies within this mixture bind to two separate isotopes on the TNFα antigen, which brings two conjugated oligonucleotides into close proximity. A DNA ligase connects the ends of the two conjugated oligonucleotides, creating a PCR template. Copies of the template can be amplified by PCR, following manufacturer's instructions. Based on fluorescent dyes, indicating amplicon, standard curves and TNFα serum levels were calculated.

### Selenop analysis by Dot blot

Selenop was detected by Dot blot. Therefore 5 μL of murine serum (1:20 diluted in distilled water) were transferred to an Amersham^TM^ Protran nitrocellulose membrane (Sigma-Aldrich/Merck) in a Dot blot apparatus (Bio-Rad Laboratories, Munich, Germany) following washing steps with TBS buffer (0.14 M NaCl, 2.7 mM KCl, 25 mM Tris, pH 7.3). Ponceau S staining (0.1 % (w/v) Ponceau S, 0.5 % (w/v) glacial acetic acid in water) was performed prior to blocking (1 h) and antibody incubation (4°C overnight, followed by 1 h RT) with 5 % (w/v) milk powder in TBST (0.1 % (v/v Tween 20, 1x TBS) and rabbit anti-Selenop/rabbit anti-mouse IgG-617 antibody (1:400; [95]). After removal of the excess antibody with TBST, goat anti-rabbit horseradish peroxidase-coupled antibody (Dako, Agilent, 1:2000) was incubated for 1 h at RT. Indirect quantification was performed with Hyperfilm^TM^ ECL (GE Healthcare Amersham, ThermoFisher Scientific) and an enhanced chemiluminescence (ECL)-based Prime Western blotting detection system (GE Healthcare, Sigma-Aldrich/Merck), incubated for 30 min. Scanned blots were quantified with Image J software (Wayne Rasband, National Institutes of Health, Bethesda, MD, USA).

### Enzyme activities

Frozen organ samples were homogenized with Tris buffer (100 mM Tris, 300 mM KCl, pH 7.6 with 0.1 % Triton X-100 (Serva, Heidelberg, Germany)). After the removal of cellular debris by centrifugation (15 min, 14,000 x g, 4°C), protein concentrations were determined by Bradford analysis (Bio-Rad Laboratories). Measurement of GPX [96], NQO1 [97], and GST [98] activities have been reported before. Briefly, GPX activity was determined in a NADPH-consuming glutathione reductase coupled assay, whereas NQO1 activity was detected by a menadione-mediated reduction of 3-(4, 5-dimethylthiazol-2-yl)-2, 5-diphenyltetrazolium bromide (MTT). GST activity was carried out using 1-chloro-2,4-dinitrobenzene (CDNB) as a substrate in the presence of reduced glutathione. All measurements were conducted in triplicates on a 96-well plate using a microplate reader (Synergy2, BioTek, Bad Friedrichshall, Germany).

### Analyses of the thyroid

Thyroid lobes were dissected from the trachea, frozen in liquid nitrogen and stored at -80°C till further use. Frozen thyroids were dropped in 10 mM Tris and immediately homogenized by rotating micropestile. Part of the homogenate was used for protein measurement using Bradford reagent (Bio-Rad Laboratories), I determination, and Dio1 activity assays. For the determination of thyroid I content, equal amounts of thyroid protein (10 μg) were mixed with ammonium persulfate (0.6 M; Sigma-Aldrich/Merck) to a total volume of 50 μL and subsequently heated up to 95°C for 1 h. After cooling, the resulting digest was further diluted (1:20) and 50 μL were transferred to a microtiter plate. Measurement of I was done by the Sandell-Kolhoff reaction in microtiter plate format, as described earlier [99]. I content was calculated to mg I per g protein.

The Dio1 activity was determined by a non-radioactive method, based on iodide-determination via Sandell-Kolthoff reaction, as described earlier [100]. In brief, 25 μg of thyroid protein homogenate was incubated for 2 h at 37°C and under constant shaking in the presence of reverse triiodothyronine (rT3; 10 μM) and 1,4-Dithiothreitol (DTT; 40 mM) in monopotassium phosphate (KPO_4_) buffer (100 mM KPO_4_, 1 mM EDTA, pH 6.8). Subsequently, released iodide was separated by ion-exchanger columns (DOWEX-50WX2, Serva) and quantified via Sandell-Kolthoff reaction. Background signal, derived from a subset of 6-n-propyl-2-thio-uracil (PTU)-inhibited reactions, was subtracted. Absolute activity was calculated from an external I standard curve, using a commercial ion chromatography standard (TraceCERT, Sigma-Aldrich/Merck).

### RNA isolation, reverse transcription, and quantitative real-time PCR

Total RNA was isolated using Trizol Reagent (Invitrogen, ThermoFisher Scientific) following the instructions of the manufacturer. After eliminating the genomic DNA using PerfeCTa DNase I (Quanta BioSciences, Beverly, MA, USA), 5 μg RNA were used for reverse transcription reaction in a final volume of 20 μL (qScript cDNA synthesis, Quanta BioSciences), generating complementary DNA (cDNA). cDNA was amplified using 1x PerfeCTa SYBR Green Supermix (Quanta BioSciences) and 250 nM primer (sequences are listed in Table 1) in a total volume of 10 μL. Real-time PCR was performed in triplicate using a CFX Connect Real-time System (Bio-Rad Laboratories) under the following conditions: 3 min at 95°C, followed by 41 cycles of 15 s at 95°C, 20 s at 60°C, and 30 s at 72°C. For quantification of mRNA levels, standard curves were taken into account to correct for differences in PCR efficiencies. Finally, expression levels were normalized to a composite factor based on the house-keeping genes Hprt and Rpl13a.

**Table 1:** Oligonucleotide sequences (5′→3′).

**Gene**	**RefSeq-ID**	**Sequence**
ATP7A, ATPase Copper Transporting Alpha	NM_001109757.2	GTCTCTGGGATGACCTGTGCT
		TCTTACTTCTGCCTTGCCAGCC
ATP7B, ATPase Copper Transporting Beta	NM_007511.2	CAGATGTCAAAGGCTCCCATTCAG
		CCAATGACGATCCACACCACC
Cp, ceruloplasmin	NM_001276248.1	GTACTACTCTGGCGTTGACCC
		TTGTCTACATCTTTCTGTCTCCCA
Ctr1, solute carrier family 31, member 1	NM_175090.4	ACCATGCCACCTCACCACCA
		GCTCCAGCCATTTCTCCAGGT
DMT1, solute carrier family 11 (proton-coupled divalent metal ion transporters), member 2	NM_001146161.1	CTCAGCCATCGCCATCAATCTC
		TTCCGCAAGCCATATTTGTCCA
Fpn, ferroportin, solute carrier family 40 (iron-regulated transporter), member 1	NM_016917.2	CTGGTGGTTCAGAATGTGTCCGT
		AGCAGACAGTAAGGACCCATCCA
Hprt1, hypoxanthine guanine phosphoribosyl transferase 1	NM_013556.2	GCAGTCCCAGCGTCGTG
		GGCCTCCCATCTCCTTCAT
IL1β, interleukin 1 beta	NM_008361.3	TTGAAGAAGAGCCCATCCTCTGTG
		TTGTTCATCTCGGAGCCTGTAGTG
IL6, interleukin 6	NM_031168.1	TCTCTGCAAGAGACTTCCATCCA
		GTCTGTTGGGAGTGGTATCCTCTG
Mt1, metallothionein 1	NM_013602.3	CTCCTGCAAGAAGAGCTGCTG
		GCACAGCACGTGCACTTGTC
Mt2, metallothionein 2	NM_008630.2	TCCTGTGCCTCCGATGGATC
		TTGCAGATGCAGCCCTGGGA
NIS, solute carrier family 5 (sodium iodide symporter), member 5	NM_053248.2	CTAGAACTGCGCTTCAGCCGA
		ACCCGGTCACTTGGTTCAGGA
Rpl13a, ribosomal protein L13a	NM_009438.5	GTTCGGCTGAAGCCTACCAG
		TTCCGTAACCTCAAGATCTGCT
Selenop, selenoprotein P	NM_001042613.1	CTCATCTATGACAGATGTGGCCGT
	AAGACTCGTGAGATTGCAGTTTCC
Slc48a1, solute carrier family 48 (heme transporter), member 1	NM_026353.4	ATTGGCCATCACCCAGCATCAG
		CTGATGTCCGCAAAGTCAGCC
Tfrc, transferrin receptor	NM_011638.4	GGCTGAAACGGAGGAGACAGA
		CTGGCTCAGCTGCTTGATGGT
TNFα, tumor necrosis factor alpha	NM_013693	CCACGTCGTAGCAAACCACC
		TACAACCCATCGGCTGGCAC
Zip 4, solute carrier family 39, member 4	NM_028064	CTCTGCAGCTGGCACCAA
		CACCAAGTCTGAACGAGAGCTTT
Zip 8, solute carrier family 39, member 8	NM _026228	CTAACGGACACATCCACTTCGA
		CCTTCAGACAGGTACATGAGCTT
Zip 14, solute carrier family 39, member 14	NM_144808	GAGCCAACTGATAATCCATTGCT
		GTCAACGGCCACATTTTCAA
ZnT1, solute carrier family 30, member 1 (Slc30a1)	NM_009579	CACGACTTACCCATTGCTCAAG
		CTTTCACCAAGTGTTTGATATCGATT
ZnT10, solute carrier family 30, member 10 (Slc30a10)	NM_001033286	ACTGGCAGTGCTACATTGACCC
		CAGCTGGCTCATCAGCTCTTC

### Western blot analysis

Liver tissue was homogenized in lysis buffer (10 mM Tris-HCl pH 7.5, 0.9 % NP-40, 0.1 % SDS, 1 mM Pefablock, protease inhibitors) using a Tissue Lyser (Qiagen, 2x2 min, 30 Hz). Homogenates were cleared by centrifugation (15 min, 14,000 x g). Protein determination was performed by Lowry assay (DC™ Protein Assay, Bio-Rad Laboratories) and samples were diluted 1:3 with 4x Laemmli sample buffer (0.25 M Tris pH 6.8, 8 % SDS, 40 % Glycerol, 0.03 % bromophenol blue), followed by denaturation for 5 min at 95°C. Proteins were loaded and separated on 15 % polyacrylamide gels and transferred to a 0.45 μm nitrocellulose membrane (Amersham™ Protran^®,^ Sigma-Aldrich/Merck) via a semi-dry blotting system (Bio-Rad Laboratories). Membranes were blocked in blocking buffer (LI-COR Bioscience, Lincoln, NE, USA; #927-40000) diluted 1:2 in PBS for 1 h at RT. Primary antibodies were diluted in blocking solution with 0.1 % Tween 20 (Merck) and incubated overnight at 4°C. Mouse anti-3-NT (Abcam, ab110282, 1:1,000), rabbit anti-glyceraldehyde-3-phosphate dehydrogenase (GAPDH; Abcam, ab37168, 1:10,000), mouse anti-FTH (Abcam, ab77127, 1:1,000) were used as primary antibodies for immunoblot detection. Secondary antibodies conjugated to IRDye® 800CW (LI-COR Bioscience; #926-32212, 1:15,000) and 680LT (LI-COR Bioscience; #926-68021, 1:15,000) were diluted in blocking solution with 0.1 % Tween 20 and incubated for 1 h at RT. Membranes were scanned using the Odyssey® CLx Imaging System (LI-COR Bioscience) and quantified with Image Studio™ (LI-COR Bioscience; v. 4.0.21). Protein levels were normalized to house-keeping gene GAPDH.

### DNA hydroxymethylation

DNA was extracted from 30 mg liver tissue via phenol/chloroform extraction. Briefly, this included lysis with cetyltrimethylammonium bromide (CTAB) buffer (pH 8.0; PanReac AppliChem GmbH, Darmstadt, Germany) using a bead ruptor 12 (Omni international Inc, Kennesaw, USA), treatment with RNase A (Promega, Madison, USA) and Proteinase K (Biolabproducts GmbH, Bebensee, Germany), extraction of DNA with phenol/chloroform/isoamyl alcohol (25:24:1 v/v, Carl Roth) and chloroform/isoamyl alcohol (24:1, Carl Roth), and precipitation with ice-cold isopropanol (99.5 %, Sigma-Aldrich/Merck) overnight. Finally, DNA was dissolved in 100 μL of diethyl dicarbonate (DEPC) treated water (Carl Roth). DNA content was measured with a NanoDrop^TM^ One (Thermo Fischer Scientific) and purity of samples was identified with the wavelength ratio A_260/280_ = 1.8–2. Aliquots of 12 μg DNA were stored at -80°C until enzymatic hydrolysis. Hydrolyzation and LC-MS/MS measurements were carried out as described in detail before [101]. Briefly, DNA was hydrolyzed using micrococcal nuclease from *Staphylococcus aureus*, bovine spleen phosphodiesterase (both Sigma-Aldrich/Merck) (incubation: 1 h, 37°C) and subsequently alkaline phosphatase (Sigma-Aldrich/Merck) (incubation: 1.5 h, 37°C). Internal standards [^15^N_2_,^13^C]-dC, mdC-D_3,_ hmdC-D_3_ (Toronto Research Chemicals, Toronto, Canada) were added before start of hydrolysis. For the measurement of mdC and dC, samples were diluted 1:20. For measurement of hmdC, samples were evaporated to dryness, proteins were removed by treatment with methanol overnight at -20°C and after removal of methanol, samples were finally taken up in a smaller volume (final dilution 1:6). HPLC-MS/MS measurements were carried out with an Agilent 1260 Infinity (Agilent Technologies) coupled to a tandem mass spectrometer (MS/MS, Agilent 6495A, Agilent Technologies). The separation was achieved using an Atlantic T3 column (2.1 x 150 mm, particle size 5 μm; Waters GmbH, Eschborn, Germany). Electrospray ionization was operated in positive mode.

### Statistics analyses

Data are given as mean. Statistical calculation was performed in Origin Pro (OriginLab, Northampton, MA, USA) using a Two-Way analysis of variance (ANOVA) with Bonferroni´s post-test. Comparisons between two groups were tested for normal distribution (Kolmogorov-Smirnov and Shapiro-Wilk test) and variances (Levene test) and subjected to either two-tailed unpaired Student´s t-test or Kruskal-Wallis ANOVA. The correlation coefficient was calculated according to Spearman (referred to as r_S_). p < 0.05 was considered statistically significant.

## Supplementary Material

Supplementary Figures

Supplementary Tables
