# Supplementary material for: Aging affects sex- and organ-specific trace element profiles in mice
Source: Aging (Albany NY). 2020 Jul 3;12(13):13762–90. doi: 10.18632/aging.103572 (PMC7377894; doi:10.18632/aging.103572)
Supplement: Supplementary Figures [file aging-12-103572-s001..pdf]

SUPPLEMENTARY FIGURES

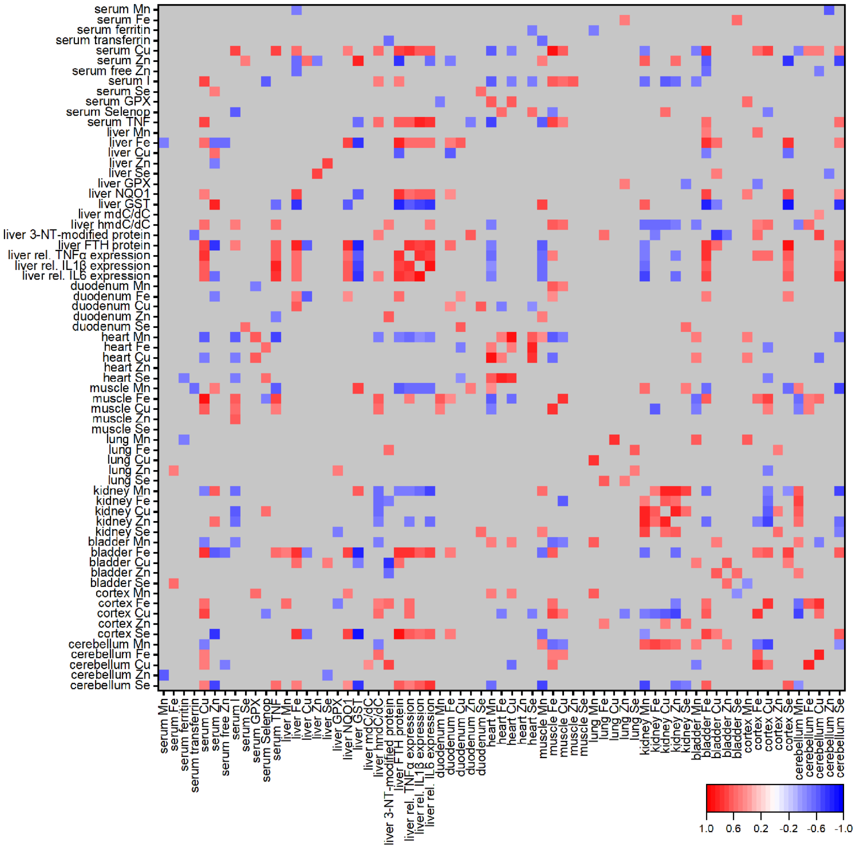

Supplementary Figure 1. Pairwise correlation coefficients among studied markers based on Spearman´s correlation. TE concentrations determined by ICP/MS and various TE-related markers of 4-5 adult (24 weeks) and old (109-114 weeks) mice of both sexes fed with a chow diet were correlated in a pairwise manner according to Spearman. Correlation coefficients varying between -1 and 1 are indicated by colored changes. Thereby, blue and red indicate significant negative and positive correlations, respectively, while grey indicates no significant correlation ($p > 0.05$).

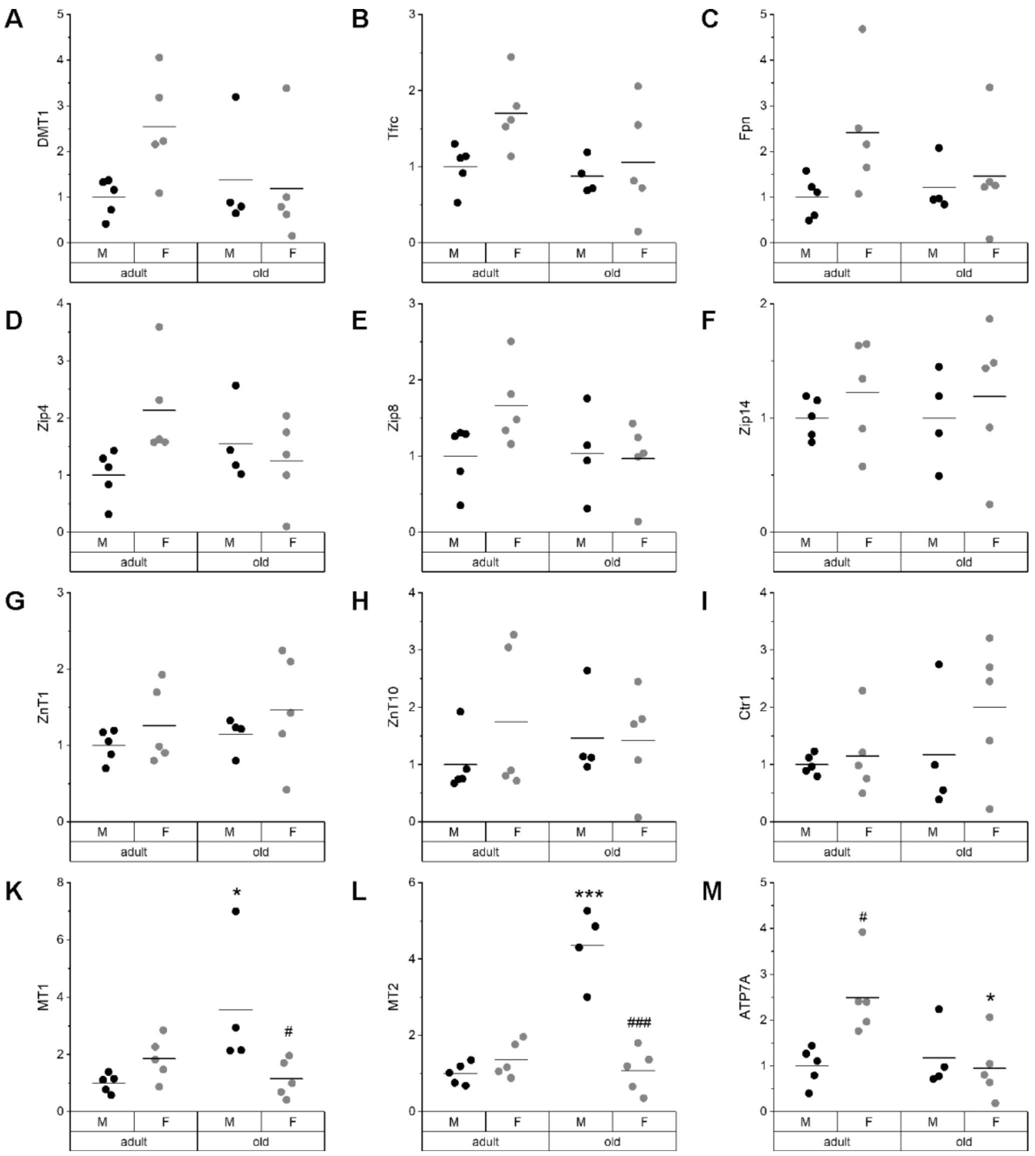

Supplementary Figure 2. Expression analysis of various TE-related genes in the duodenum. Relative expression levels of TE-related genes in the duodenum of 4-5 adult (24 weeks) and old (109-114 weeks) mice of both sexes fed with a chow diet *ad libitum*. Expression levels were normalized to a composite factor based on the house-keeping genes Hprt and Rpl13a, finally variances are expressed as fold change compared to male adults. Statistical testing based on Two-Way ANOVA and post hoc analysis using Bonferroni´s test with * $p < 0.05$, *** $p < 0.001$ vs. adult and $^{\#}$ $p < 0.05$, $^{\#\#\#}$ $p < 0.001$ vs. male.

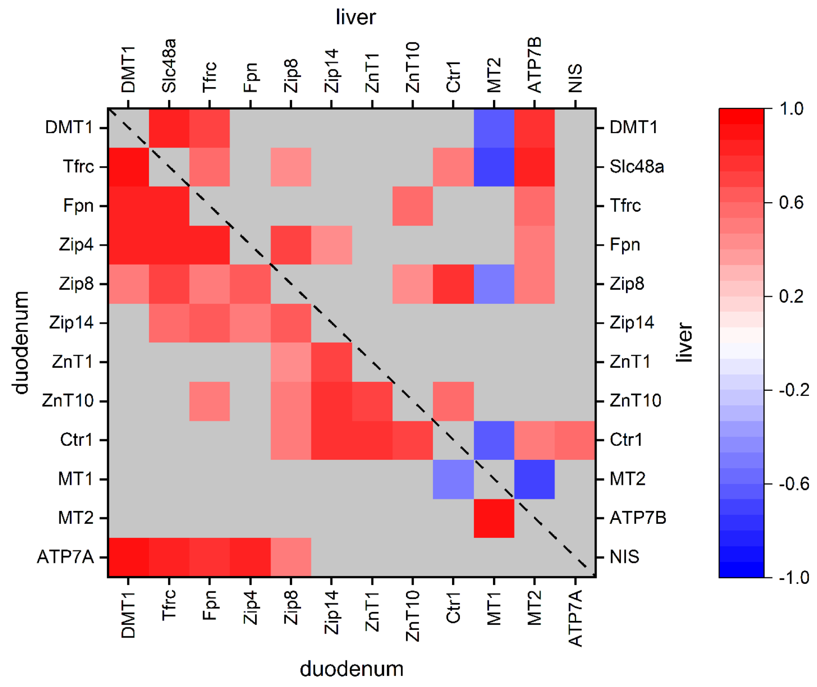

Supplementary Figure 3. Pairwise correlation coefficients among TE-related genes in duodenum and liver based on Spearman´s correlation. Relative expression levels of transporters or binding proteins for the TEs Cu, Fe, Mn, or Zn in the duodenum and liver of 4-5 adult (24 weeks) and old (109-114 weeks) mice of both sexes fed with a chow diet *ad libitum* were assessed by qRT-PCR. Expression levels were normalized to a composite factor based on the house-keeping genes Hprt and Rpl13a and correlated according to Spearman. Correlation coefficients vary between -1 and 1, indicated by colored changes. Thereby, blue and red indicate significant negative and positive correlations, respectively, while grey indicates no significant correlations ($p > 0.05$).

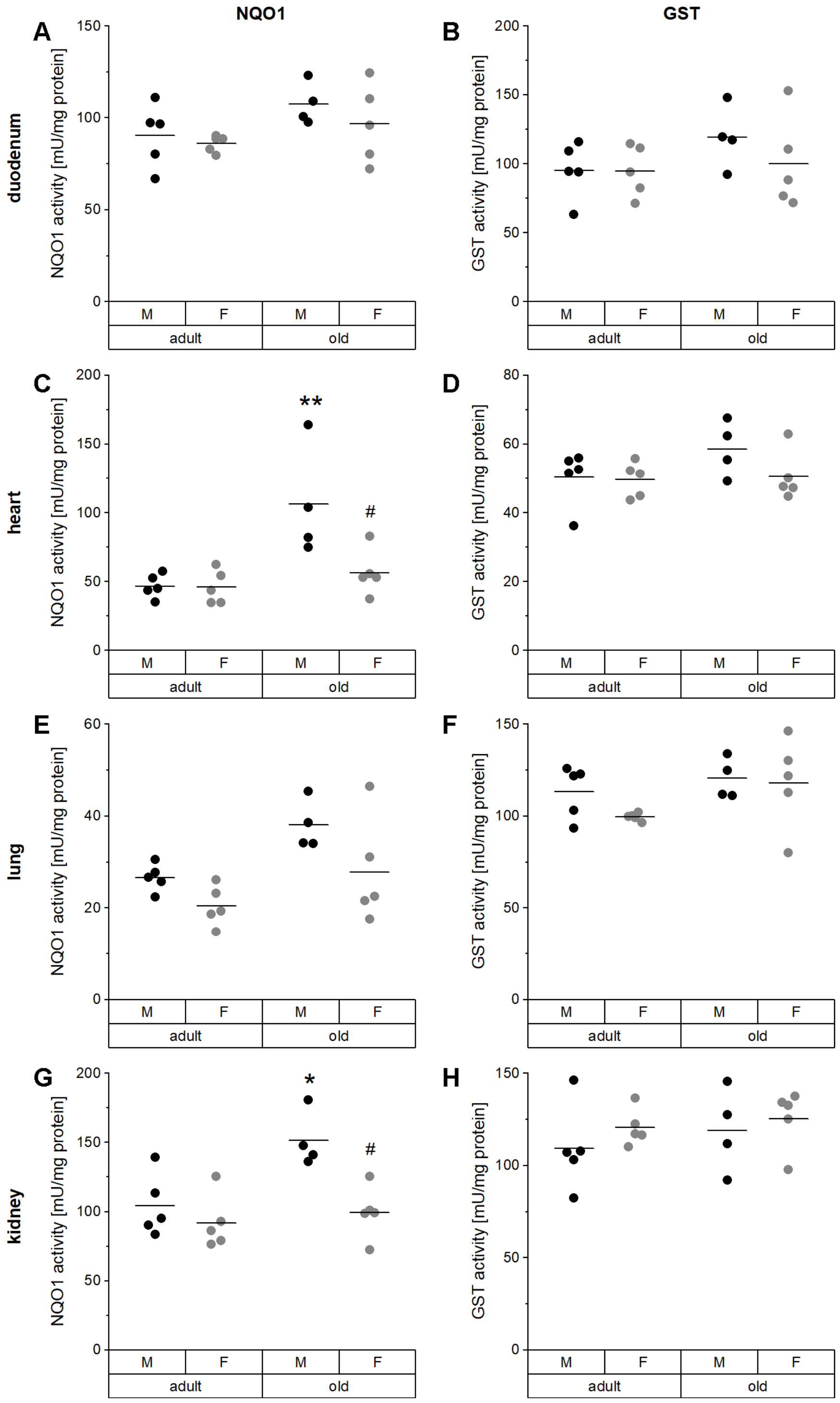

Supplementary Figure 4. Enzyme activities in various organs. The enzyme activity of Nrf2 targets NQO1 (**A**, **C**, **E**, **G**) and GST (**B**, **D**, **F**, **H**) were determined by activity assay. Samples were either organ samples of duodenum (**A**, **B**), heart (**C**, **D**), lung (**E**, **F**), or kidney (**G**, **H**) of 4-5 adult (24 weeks) and old (109-114 weeks) mice of both sexes. Statistical testing based on Two-Way ANOVA and post hoc analysis using Bonferroni´s test with ** $p < 0.01$ vs. adult and $^{\#}$ $p < 0.05$ vs. male.

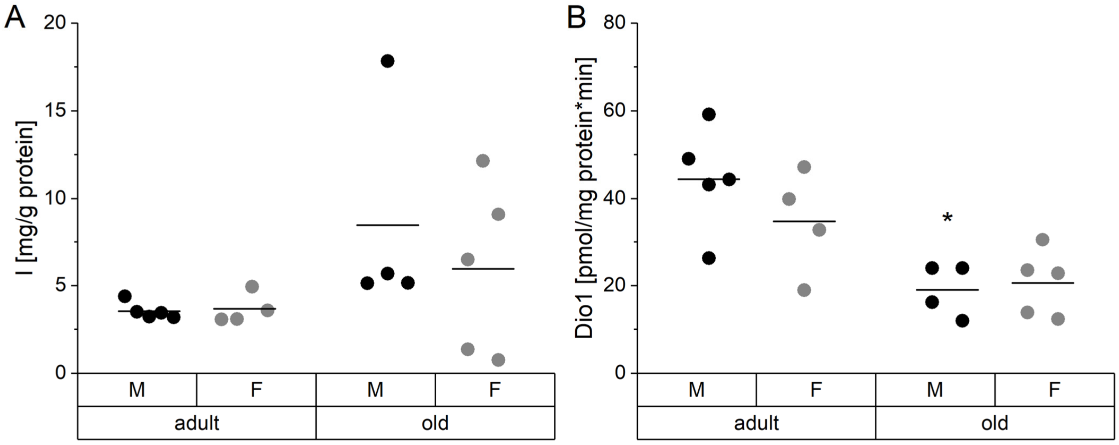

Supplementary Figure 5. I content and deiodinase activity of the thyroid. Thyroidal I content (**A**) and I-releasing enzyme activity of Dio1 (**B**) were determined by Sandell-Kolthoff-based I measurement in ammonium persulfate-digested organ and non-radioactive activity assay, respectively. Samples were thyroids of 4-5 adult (24 weeks) and old (109-114 weeks) mice of both sexes. I content is calculated to mg I per g protein. Statistical testing based on Two-Way ANOVA and post hoc analysis using Bonferroni´s test with * $p < 0.05$ vs. adult.
