# Supplementary material for: Aging affects sex- and organ-specific trace element profiles in mice
Source: Aging (Albany NY). 2020 Jul 3;12(13):13762–90. doi: 10.18632/aging.103572 (PMC7377894; doi:10.18632/aging.103572)
Supplement: Supplementary Tables [file aging-12-103572-s002..pdf]

SUPPLEMENTARY TABLES

Supplementary Table 1. Mouse data.

general features	adult ♂	adult ♀	elderly ♂	elderly ♀	age	sex	age X sex
body weight [g]	31.9 ± 1.8	25.7 ± 1.3	31.7 ± 1.4	29.9 ± 1.6	*	***	**
relative liver weight [mg/g BW]	42.9 ± 14.4	48.6 ± 5.6	41.8 ± 5.2	53.6 ± 6.9	n.s.	0.052	n.s.
relative spleen weight [mg/g BW]	2.4 ± 0.2	3.6 ± 0.5	2.5 ± 0.7	8.8 ± 7.0	n.s.	*	n.s.
relative heart weight [mg/g BW]	4.5 ± 0.3	4.4 ± 0.2	5.1 ± 1.1	6.0 ± 0.6	**	n.s.	n.s.
relative kidney weight [mg/g BW]	11.7 ± 0.3	11.4 ± 0.6	13.1 ± 2.8	16.4 ± 2.1	**	0.078	*
relative cortex weight [mg/g BW]	9.5 ± 0.7	11.9 ± 0.9	10.0 ± 0.6	10.3 ± 2.6	n.s.	0.069	n.s.
relative cerebellum weight [mg/g BW]	3.0 ± 1.5	5.0 ± 0.3	4.2 ± 0.3	5.8 ± 3.3	n.s.	0.061	n.s.
relative length of small intestine [cm/g BW]	1.07 ± 0.10	1.27 ± 0.10	1.14 ± 0.06	1.05 ± 0.03	0.074	0.070	**
relative length of colon [cm/g BW]	0.26 ± 0.02	0.30 ± 0.02	0.25 ± 0.02	0.29 ± 0.02	n.s.	**	n.s.

Data represent mean ± standard deviation for various clinical parameters of 4-5 adult (24 weeks) and old (109-114 weeks) mice of both sexes fed with chow diet. Statistical testing based on Two-Way ANOVA and post hoc analysis using Bonferroni´s test with * $p < 0.05$, ** $p < 0.01$, *** $p < 0.001$, n.s. non-significant, $p > 0.1$. Trends with $p < 0.1$ were indicated. BW = body weight.

Supplementary Table 2. TE concentrations in various organs of C57BL/6Jrj mice.

organ	TE	unit	adult ♂	adult ♀	elderly ♂	elderly ♀	age	sex	age X sex
serum	Cu	[mg/L]	0.46 ± 0.07	0.44 ± 0.02	0.55 ± 0.03	0.99 ± 0.26	***	**	**
	I	[µg/L]	97.09 ± 18.63	87.53 ± 17.01	107.27 ± 26.72	130.47 ± 26.47	*	n.s.	n.s.
	Fe	[mg/L]	2.15 ± 0.74	3.12 ± 1.97	1.87 ± 0.76	2.04 ± 0.41	n.s.	n.s.	n.s.
	Mn	[µg/L]	3.54 ± 0.56	3.14 ± 0.47	3.30 ± 0.79	2.93 ± 0.69	n.s.	n.s.	n.s.
	Se	[mg/L]	0.34 ± 0.03	0.30 ± 0.02	0.33 ± 0.05	0.32 ± 0.08	n.s.	n.s.	n.s.
	Zn	[mg/L]	0.91 ± 0.14	0.69 ± 0.10	0.72 ± 0.07	0.68 ± 0.02	*	**	*
liver	Cu	[mg/kg]	4.97 ± 0.58	4.22 ± 0.58	4.24 ± 0.31	3.97 ± 0.26	*	*	n.s.
	Fe	[mg/kg]	89.40 ± 1.82	137.72 ± 33.52	121.75 ± 38.01	217.20 ± 91.85	*	*	n.s.
	Mn	[mg/kg]	0.97 ± 0.09	1.11 ± 0.25	1.32 ± 0.29	1.14 ± 0.28	n.s.	n.s.	n.s.
	Se	[mg/kg]	1.11 ± 0.09	1.15 ± 0.28	1.10 ± 0.11	1.12 ± 0.16	n.s.	n.s.	n.s.
	Zn	[mg/kg]	26.10 ± 1.86	28.68 ± 4.95	26.80 ± 2.00	27.12 ± 2.54	n.s.	n.s.	n.s.
duo-denum	Cu	[mg/kg]	1.86 ± 0.07	1.89 ± 0.10	1.91 ± 0.21	2.14 ± 0.25	0.085	n.s.	n.s.
	Fe	[mg/kg]	47.21 ± 7.80	52.75 ± 15.41	49.80 ± 4.09	66.78 ± 9.00	0.098	*	n.s.
	Mn	[mg/kg]	1.67 ± 0.42	1.36 ± 0.21	1.62 ± 0.31	1.64 ± 0.34	n.s.	n.s.	n.s.
	Se	[mg/kg]	0.46 ± 0.04	0.45 ± 0.02	0.45 ± 0.04	0.43 ± 0.03	n.s.	n.s.	n.s.
	Zn	[mg/kg]	20.92 ± 0.96	21.16 ± 4.24	22.28 ± 2.57	19.95 ± 1.37	n.s.	n.s.	n.s.
heart	Cu	[mg/kg]	7.79 ± 0.94	8.86 ± 1.76	7.11 ± 0.46	7.01 ± 0.74	*	n.s.	n.s.
	Fe	[mg/kg]	149.96 ± 13.23	164.38 ± 51.65	137.60 ± 8.15	130.74 ± 19.36	n.s.	n.s.	n.s.
	Mn	[mg/kg]	0.87 ± 0.09	1.03 ± 0.21	0.83 ± 0.08	0.63 ± 0.18	**	n.s.	*
	Se	[mg/kg]	0.40 ± 0.03	0.45 ± 0.10	0.37 ± 0.04	0.38 ± 0.05	n.s.	n.s.	n.s.
	Zn	[mg/kg]	18.08 ± 3.54	20.16 ± 5.09	20.73 ± 5.96	16.98 ± 1.72	n.s.	n.s.	n.s.
muscle	Cu	[mg/kg]	1.07 ± 0.14	0.97 ± 0.05	1.09 ± 0.06	1.19 ± 0.12	*	n.s.	0.053
	Fe	[mg/kg]	19.69 ± 1.20	16.65 ± 2.56	20.72 ± 1.19	27.55 ± 4.72	***	n.s.	**
	Mn	[mg/kg]	0.20 ± 0.03	0.18 ± 0.01	0.20 ± 0.03	0.13 ± 0.02	0.060	**	0.060
	Se	[mg/kg]	0.22 ± 0.02	0.21 ± 0.01	0.21 ± 0.02	0.21 ± 0.02	n.s.	n.s.	n.s.

	Zn	[mg/kg]	3.27 ± 1.23	3.75 ± 1.20	3.35 ± 1.34	4.70 ± 1.60	n.s.	n.s.	n.s.
lung	Cu	[mg/kg]	2.53 ± 0.23	2.76 ± 0.22	2.82 ± 0.36	3.52 ± 1.78	n.s.	n.s.	n.s.
	Fe	[mg/kg]	111.58 ± 11.87	125.99 ± 29.20	133.62 ± 29.05	115.60 ± 22.28	n.s.	n.s.	n.s.
	Mn	[mg/kg]	0.24 ± 0.03	0.33 ± 0.12	0.32 ± 0.09	0.25 ± 0.07	n.s.	n.s.	*
	Se	[mg/kg]	0.46 ± 0.03	0.49 ± 0.04	0.50 ± 0.05	0.45 ± 0.05	n.s.	n.s.	0.093
	Zn	[mg/kg]	17.77 ± 2.11	19.13 ± 1.86	17.02 ± 1.33	17.39 ± 2.25	n.s.	n.s.	n.s.
kidney	Cu	[mg/kg]	4.19 ± 0.34	4.28 ± 0.23	3.54 ± 0.65	3.73 ± 0.53	*	n.s.	n.s.
	Fe	[mg/kg]	94.23 ± 11.19	116.32 ± 18.21	77.00 ± 10.66	102.76 ± 39.65	n.s.	*	n.s.
	Mn	[mg/kg]	1.45 ± 0.13	1.31 ± 0.09	1.10 ± 0.33	1.04 ± 0.23	**	n.s.	n.s.
	Se	[mg/kg]	1.54 ± 0.16	1.42 ± 0.10	1.66 ± 0.48	1.36 ± 0.09	n.s.	0.080	n.s.
	Zn	[mg/kg]	19.05 ± 2.20	17.70 ± 0.97	15.10 ± 1.89	15.76 ± 1.58	**	n.s.	n.s.
bladder	Cu	[mg/kg]	0.89 ± 0.09	1.19 ± 0.12	0.89 ± 0.08	1.08 ± 0.17	n.s.	***	n.s.
	Fe	[mg/kg]	13.80 ± 3.77	21.98 ± 2.14	25.07 ± 5.35	43.46 ± 5.26	***	***	*
	Mn	[mg/kg]	0.12 ± 0.01	0.18 ± 0.04	0.13 ± 0.02	0.12 ± 0.02	*	0.075	**
	Se	[mg/kg]	0.33 ± 0.02	0.34 ± 0.01	0.32 ± 0.02	0.31 ± 0.06	n.s.	n.s.	n.s.
	Zn	[mg/kg]	31.97 ± 2.94	35.23 ± 4.43	29.45 ± 2.63	28.13 ± 5.69	*	n.s.	n.s.
cortex	Cu	[mg/kg]	3.86 ± 0.09	3.84 ± 0.08	5.26 ± 0.39	5.16 ± 0.48	***	n.s.	n.s.
	Fe	[mg/kg]	19.25 ± 0.46	19.24 ± 0.74	26.99 ± 0.81	23.02 ± 2.25	***	**	**
	Mn	[mg/kg]	0.42 ± 0.01	0.44 ± 0.01	0.43 ± 0.02	0.41 ± 0.08	n.s.	n.s.	n.s.
	Se	[mg/kg]	0.18 ± 0.00	0.21 ± 0.01	0.20 ± 0.01	0.22 ± 0.01	**	***	n.s.
	Zn	[mg/kg]	16.22 ± 0.29	16.15 ± 0.17	16.37 ± 0.29	15.63 ± 1.63	n.s.	n.s.	n.s.
cere-bellum	Cu	[mg/kg]	5.16 ± 0.27	4.88 ± 0.30	7.20 ± 0.80	6.06 ± 1.08	***	*	n.s.
	Fe	[mg/kg]	21.69 ± 1.87	21.30 ± 1.94	24.64 ± 1.01	23.91 ± 2.52	**	n.s.	n.s.
	Mn	[mg/kg]	0.56 ± 0.05	0.59 ± 0.02	0.52 ± 0.03	0.49 ± 0.06	**	n.s.	n.s.
	Se	[mg/kg]	0.21 ± 0.01	0.22 ± 0.01	0.22 ± 0.01	0.23 ± 0.01	**	**	n.s.
	Zn	[mg/kg]	13.84 ± 0.98	14.02 ± 2.05	13.26 ± 0.42	14.70 ± 1.44	n.s.	n.s.	n.s.

Values are shown as mean ± standard deviation of TE concentrations in various organs of 4-5 adult (24 weeks) and old (109-114 weeks) mice of both sexes receiving chow diet. Multielement analysis for Cu, I (only serum), Fe, Mn, Se, and Zn rely on ICP-MS/MS measurements. Statistical testing based on Two-Way ANOVA and post hoc analysis using Bonferroni´s test with * $p < 0.05$, ** $p < 0.01$, *** $p < 0.001$, n.s. non-significant. Trends with $p < 0.1$ were indicated.

Supplementary Table 3. Correlations among serum marker of C57BL/6Jrj mice.

serum parameters		Mn	I	Cu	Fe	ferritin	transferrin	Se	GPX*	Selenop	Zn	free Zn	TNFα
Mn	r_S		-0.199	-0.271	0.331	-0.013	-0.146	-0.115	0.143	0.115	0.245	0.354	-0.003
	p-value		0.428	0.276	0.179	0.958	0.565	0.651	0.570	0.672	0.328	0.150	0.990
I	r_S	-0.199		0.701	0.315	0.302	0.368	0.077	-0.288	-0.662	-0.360	-0.018	0.430
	p-value	0.428		0.001	0.203	0.223	0.132	0.760	0.247	0.005	0.142	0.945	0.075
Cu	r_S	-0.271	0.701		-0.207	0.020	0.276	0.222	-0.439	-0.453	-0.240	-0.276	0.692
	p-value	0.276	0.001		0.409	0.938	0.268	0.376	0.069	0.078	0.336	0.268	0.001
Fe	r_S	0.331	0.315	-0.207		0.410	0.069	-0.152	-0.079	-0.003	-0.220	0.112	-0.011
	p-value	0.179	0.203	0.409		0.091	0.785	0.548	0.754	0.991	0.381	0.657	0.964
ferritin	r_S	-0.013	0.302	0.020	0.41		0.067	0.036	-0.379	-0.324	0.034	0.236	0.152
	p-value	0.958	0.223	0.938	0.091		0.791	0.887	0.121	0.222	0.893	0.345	0.548
transferrin	r_S	-0.146	0.368	0.276	0.069	0.067		-0.201	-0.032	-0.406	-0.129	0.137	0.286
	p-value	0.565	0.132	0.268	0.785	0.791		0.423	0.900	0.119	0.610	0.587	0.250
Se	r_S	-0.115	0.077	0.222	-0.152	0.036	-0.201		-0.042	-0.368	0.509	-0.067	0.075
	p-value	0.651	0.760	0.376	0.548	0.887	0.423		0.868	0.161	0.031	0.791	0.766
GPX*	r_S	0.143	-0.288	-0.439	-0.079	-0.379	-0.032	-0.042		0.268	0.123	0.428	-0.317
	p-value	0.570	0.247	0.069	0.754	0.121	0.900	0.868		0.316	0.627	0.076	0.200
Selenop	r_S	0.115	-0.662	-0.453	-0.003	-0.324	-0.406	-0.368	0.268		-0.179	0.082	-0.071
	p-value	0.672	0.005	0.078	0.991	0.222	0.119	0.161	0.316		0.506	0.762	0.795
Zn	r_S	0.245	-0.360	-0.240	-0.220	0.034	-0.129	0.509	0.123	-0.179		0.375	-0.209
	p-value	0.328	0.142	0.336	0.381	0.893	0.610	0.031	0.627	0.506		0.126	0.404
free Zn	r_S	0.354	-0.018	-0.276	0.112	0.236	0.137	-0.067	0.428	0.082	0.375		-0.082
	p-value	0.150	0.945	0.268	0.657	0.345	0.587	0.791	0.076	0.762	0.126		0.748
TNFα	r_S	-0.003	0.430	0.692	-0.011	0.152	0.286	0.075	-0.317	-0.071	-0.209	-0.082	
	p-value	0.990	0.075	0.001	0.964	0.548	0.250	0.766	0.200	0.795	0.404	0.748	

Explorative Spearman´s correlation coefficient (r_S) analysis was performed for all parameters analyzed in sera of 19 C57BL/6Jrj mice. Investigated parameters included levels of trace elements other markers, as well as enzyme activity (indicated with *). Significance is reflected by p-values. Correlations with high correlation coefficient (r_S>0.5) and significant p-value are indicated in bold.

Supplementary Table 4. Correlations among liver markers of C57BL/6Jrj mice.

liver parameter		Mn	Fe	Cu	Zn	Se	GPX*	TNFα#	IL1β#	IL6#	mdC/dC	hmdC/dC	3-NT-modified protein	NQO1*	GST*	FTH
**Mn**	r_S		0.445	-0.089	0.371	0.304	-0.357	0.322	0.362	0.393	0.086	0.087	0.226	0.424	-0.296	0.291
	p-value		0.056	0.717	0.118	0.205	0.133	0.179	0.127	0.096	0.725	0.724	0.384	0.071	0.219	0.241
**Fe**	r_S	0.445		-0.389	0.363	0.113	-0.079	**0.576**	**0.544**	**0.541**	0.224	-0.037	-0.337	**0.710**	**-0.741**	**0.811**
	p-value	0.056		0.100	0.127	0.644	0.748	**0.010**	**0.016**	**0.017**	0.356	0.881	0.185	**0.001**	**2.839x10^{-4}**	**4.447x10^{-5}**
**Cu**	r_S	-0.089	-0.389		0.254	0.426	-0.279	-0.444	-0.384	-0.321	-0.383	-0.321	-0.137	-0.452	0.429	**-0.612**
	p-value	0.717	0.100		0.293	0.069	0.247	0.057	0.105	0.180	0.105	0.180	0.599	0.052	0.067	**0.007**
**Zn**	r_S	0.371	0.363	0.254		0.677	0.176	0.034	0.200	0.293	-0.071	-0.284	-0.360	0.080	-0.414	0.145
	p-value	0.118	0.127	0.293		0.001	0.470	0.889	0.411	0.223	0.772	0.238	0.156	0.745	0.078	0.567
**Se**	r_S	0.304	0.113	0.426	0.677		-0.067	-0.132	-0.025	0.156	-0.246	-0.097	-0.187	-0.279	-0.197	-0.034
	p-value	0.205	0.644	0.069	0.001		0.786	0.591	0.920	0.523	0.310	0.694	0.473	0.247	0.419	0.893
**GPX***	r_S	-0.357	-0.079	-0.279	0.176	-0.067		-0.167	-0.039	-0.023	0.090	-0.204	-0.466	-0.054	-0.033	0.090
	p-value	0.133	0.748	0.247	0.470	0.786		0.495	0.875	0.926	0.716	0.403	0.060	0.825	0.892	0.723
**TNFα#**	r_S	0.322	**0.576**	-0.444	0.034	-0.132	-0.167		**0.747**	**0.726**	0.371	0.326	-0.091	**0.582**	**-0.663**	**0.752**
	p-value	0.179	**0.010**	0.057	0.889	0.591	0.495		**2.353x10^{-4}**	**4.292x10^{-4}**	0.118	0.173	0.729	**0.009**	**0.002**	**3.160x10^{-4}**
**IL1β#**	r_S	0.362	**0.544**	-0.384	0.200	-0.025	-0.039	**0.747**		**0.902**	0.134	0.423	-0.076	**0.614**	**-0.691**	**0.701**
	p-value	0.127	**0.016**	0.105	0.411	0.920	0.875	**2.353x10^{-4}**		**1.342x10^{-7}**	0.584	0.071	0.772	**0.005**	**0.001**	**0.001**
**IL6#**	r_S	0.393	**0.541**	-0.321	0.293	0.156	-0.023	**0.726**	**0.902**		0.004	**0.514**	-0.176	**0.646**	**-0.744**	**0.744**
	p-value	0.096	**0.017**	0.180	0.223	0.523	0.926	**4.292x10^{-4}**	**1.342x10^{-7}**		0.986	**0.024**	0.498	**0.003**	**2.611x10^{-4}**	**3.992x10^{-4}**
**mdC/dC**	r_S	0.086	0.224	-0.383	-0.071	-0.246	0.090	0.371	0.134	0.004		-0.140	-0.113	0.181	-0.033	0.223
	p-value	0.725	0.356	0.105	0.772	0.310	0.716	0.118	0.584	0.986		0.566	0.666	0.459	0.892	0.374
**hmdC/dC**	r_S	0.087	-0.037	-0.321	-0.284	-0.097	-0.204	0.326	0.423	**0.514**	-0.140		**0.527**	0.137	-0.181	0.183
	p-value	0.724	0.881	0.180	0.238	0.694	0.403	0.173	0.071	**0.024**	0.566		**0.030**	0.576	0.459	0.468
**3-NT-modified protein**	r_S	0.226	-0.337	-0.137	-0.360	-0.187	-0.466	-0.091	-0.076	-0.176	-0.113	**0.527**		-0.309	0.395	-0.426
	p-value	0.384	0.185	0.599	0.156	0.473	0.060	0.729	0.772	0.498	0.666	**0.030**		0.228	0.117	0.088
**NQO1***	r_S	0.424	**0.710**	-0.452	0.080	-0.279	-0.054	**0.582**	**0.614**	**0.646**	0.181	0.137	-0.309		**-0.633**	**0.759**
	p-value	0.071	**0.001**	0.052	0.745	0.247	0.825	**0.009**	**0.005**	**0.003**	0.459	0.576	0.228		**0.004**	**2.636x10^{-4}**
**GST***	r_S	-0.296	**-0.741**	0.429	-0.414	-0.197	-0.033	**-0.663**	**-0.691**	**-0.744**	-0.033	-0.181	0.395	**-0.633**		**-0.851**
	p-value	0.219	**2.839x10^{-4}**	0.067	0.078	0.419	0.892	**0.002**	**0.001**	**2.611x10^{-4}**	0.892	0.459	0.117	**0.004**		**7.422x10^{-6}**
**FTH**	r_S	0.291	**0.811**	**-0.612**	0.145	-0.034	0.090	**0.752**	**0.701**	**0.744**	0.223	0.183	-0.426	**0.759**	**-0.851**	
	p-value	0.241	**4.447x10^{-5}**	**0.007**	0.567	0.893	0.723	**3.160x10^{-4}**	**0.001**	**3.992x10^{-4}**	0.374	0.468	0.088	**2.636x10^{-4}**	**7.422x10^{-6}**	

Explorative Spearman´s correlation coefficient (r_S) analysis was performed for all analyzed parameters in liver of 19 C57BL/6Jrj mice. Investigated parameters included levels of trace elements, functional or aging marker, next to enzyme activity and relative expression levels (indicated with * and #, respectively). Significance is reflected by p-values. Correlations with high correlation coefficient (r_S>0.5) and significant p-value are indicated in bold.
